# Machine Learning of Patient Characteristics to Predict Admission Outcomes in the Undiagnosed Diseases Network

**DOI:** 10.1001/jamanetworkopen.2020.36220

**Published:** 2021-02-25

**Authors:** Hadi Amiri, Isaac S. Kohane

**Affiliations:** 1 Department of Biomedical Informatics, Harvard University, Boston, Massachusetts; 2 Department of Computer Science, University of Massachusetts, Lowell

## Abstract

**Importance:**

The Undiagnosed Diseases Network (UDN) is a national network that evaluates individual patients whose signs and symptoms have been refractory to diagnosis. Providing reliable estimates of admission outcomes may assist clinical evaluators to distinguish, prioritize, and accelerate admission to the UDN for patients with undiagnosed diseases.

**Objective:**

To develop computational models that effectively predict admission outcomes for applicants seeking UDN evaluation and to rank the applications based on the likelihood of patient admission to the UDN.

**Design, Setting, and Participants:**

This prognostic study included all applications submitted to the UDN from July 2014 to June 2019, with 1209 applications accepted and 1212 applications not accepted. The main inclusion criterion was an undiagnosed condition despite thorough evaluation by a health care professional; the main exclusion criteria were a diagnosis that explained the objective findings or a review of the records that suggested a diagnosis. A classifier was trained using information extracted from application forms, referral letters from health care professionals, and semantic similarity between referral letters and textual description of known mendelian disorders. The admission labels were provided by the case review committee of the UDN. In addition to retrospective analysis, the classifier was prospectively tested on another 288 applications that were not evaluated at the time of classifier development.

**Main Outcomes and Measures:**

The primary outcomes were whether a patient was accepted or not accepted to the UDN and application order ranked based on likelihood of admission. The performance of the classifier was assessed by comparing its predictions against the UDN admission outcomes and by measuring improvement in the mean processing time for accepted applications.

**Results:**

The best classifier obtained sensitivity of 0.843, specificity of 0.738, and area under the receiver operating characteristic curve of 0.844 for predicting admission outcomes among 1212 accepted and 1210 not accepted applications. In addition, the classifier can decrease the current mean (SD) UDN processing time for accepted applications from 3.29 (3.17) months to 1.05 (3.82) months (68% improvement) by ordering applications based on their likelihood of acceptance.

**Conclusions and Relevance:**

A classification system was developed that may assist clinical evaluators to distinguish, prioritize, and accelerate admission to the UDN for patients with undiagnosed diseases. Accelerating the admission process may improve the diagnostic journeys for these patients and serve as a model for partial automation of triaging or referral for other resource-constrained applications. Such classification models make explicit some of the considerations that currently inform the use of whole-genome sequencing for undiagnosed disease and thereby invite a broader discussion in the clinical genetics community.

## Key Points

**Question:**

Can machine learning algorithms reproduce the performance of clinical experts in determining whether to accept patients to the Undiagnosed Diseases Network for extensive genome-scale evaluation?

**Findings:**

This prognostic study developed a machine learning model using 2421 patient applications and evaluated the model through retrospective and prospective validation. The area under the receiver operating characteristic curve obtained for predicting admission outcomes suggested that the admission process for accepted applications may be accelerated by up to 68% using the developed machine learning model.

**Meaning:**

Findings of this study suggest that the use of machine learning assistance to prioritize the evaluation of patients with undiagnosed diseases is feasible and may increase the number of applications processed in a given time frame.

## Teaser

This prognostic study evaluates the ability of a machine learning model to predict admission outcomes for applicants seeking genome-scale evaluation by the Undiagnosed Diseases Network.

## Introduction

Rare and undiagnosed diseases are paradigmatic for the era of precision medicine. Although there is no unique definition for such diseases,^1^ a disease is typically considered rare if its prevalence is less than 1 per 1250 in the US,^2^ per 2000 in Europe,^3^ and per 2500 and 10 000 in Japan and Australia,^4^ respectively. Many undiagnosed diseases are likely to include a genetic component to their pathogenesis, and yet patients will find themselves on a protracted journey from one specialist to another without diagnosis even in this era of genomic sequencing.^5,6^ The onset in about half of undiagnosed diseases occurs at birth or during infancy, and these diseases are often associated with premature mortality and disability that is present throughout a patient’s life.^1,7,8,9^ According to the Office of Rare Disease Research at the National Institutes of Health, approximately 6% of the inquiries made to the Genetic and Rare Disease Information Center are in reference to an undiagnosed disease.^10^ In addition, although a large number of rare diseases exist (>7000 different types^10^), only a small fraction of these diseases (approximately 350 rare diseases) affect more than 80% of all patients with rare diseases.^11^

**Figure 1.**
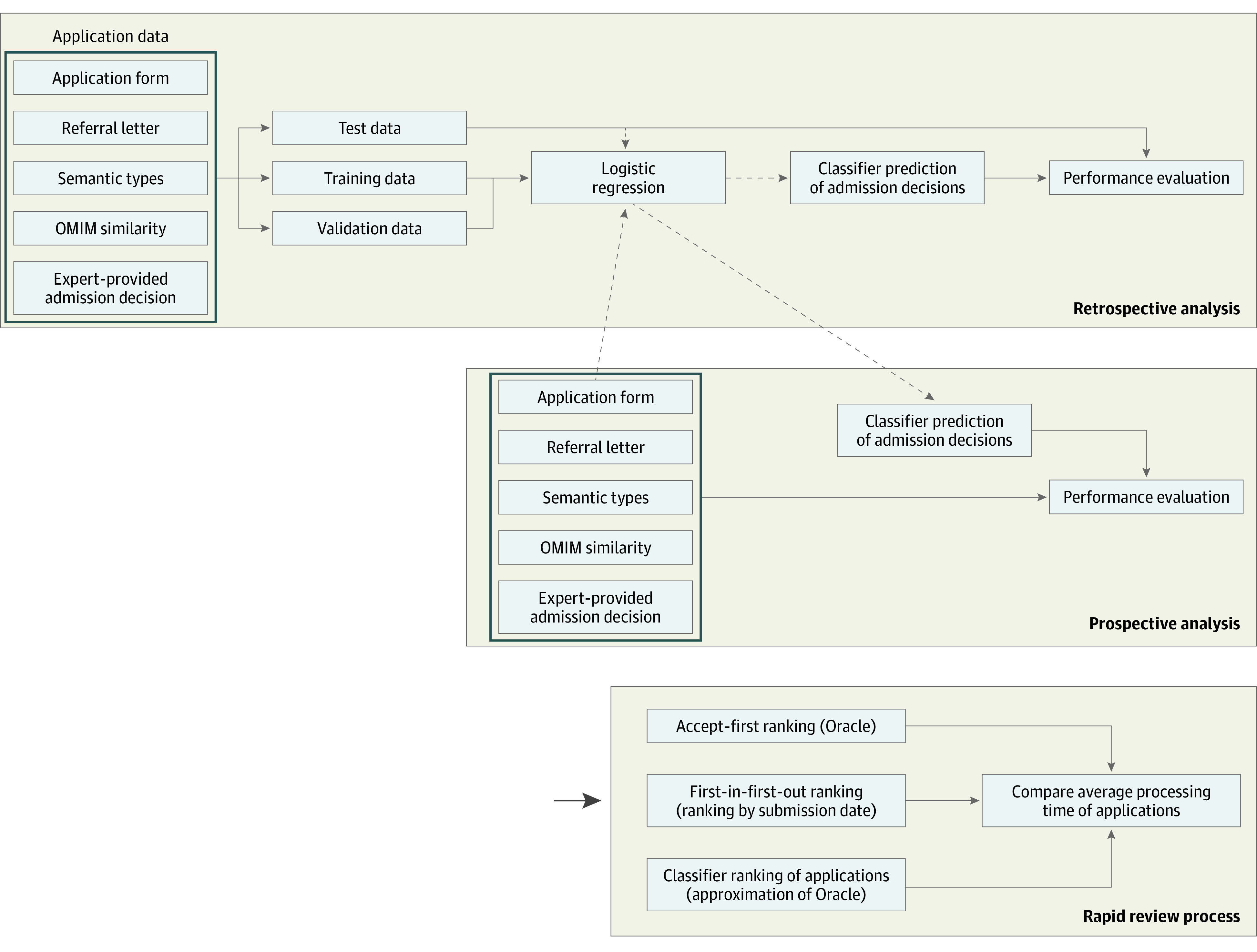
Analytic Flow for Predicting Undiagnosed Diseases Network (UDN) Application Outcomes in Retrospective and Prospective Studies and Ranking Applications for the UDN Evaluation Process OMIM represents the Online Mendelian Inheritance in Man database.

The National Institutes of Health established the Undiagnosed Diseases Network (UDN)^12,13^ to facilitate research on undiagnosed and rare diseases. The UDN^14^ is a network of 12 clinical sites. Application to the UDN is open to all individuals who complete the application form and submit a referral letter from a health care professional. A committee of experts in a review session reviews each UDN application and makes admission decisions. Currently, the UDN receives a mean (SD) of 55.0 (19.7) applications per month, and the mean (SD) processing time of applications, that is, the difference between application submission and review dates, is 3.3 (3.2) months for patients accepted (accepted applications) and 4.7 (4.9) months for those not accepted (not accepted applications) for further evaluation by UDN clinicians and investigators. Here, we developed computational models to determine how well these algorithms could simulate the outcome of the UDN expert committee using only the individual patient’s application materials and reference materials about rare diseases. Our models were designed to predict UDN application outcomes and thereby rank applications based on their likelihood of acceptance to the UDN evaluation process. The analytic flow of this investigation is depicted in Figure 1.

## Methods

We collected application materials from all the UDN sites to constitute a data set containing 2421 UDN applications that had been submitted to the UDN from July 2014 to June 2019 (eAppendix 2 in the Supplement). The data set had 1209 accepted applications and 1212 not accepted applications. The admission outcomes were provided by the case review committee of the UDN—a group of clinical experts who critically discuss and review each application. The main inclusion criteria were that the applicant should have a condition that remained undiagnosed despite thorough evaluation by a health care professional and had at least 1 objective finding pertinent to the phenotype for which the application was submitted. The main exclusion criteria were that the applicant received a diagnosis that explained the objective findings or a review of the records suggested a diagnosis and further evaluation by the UDN was deemed unnecessary. Each application in the data set had all the following information: (1) an application form containing demographic characteristic information; (2) an official referral letter signed by a health care professional summarizing the applicant’s medical problems, previous diagnoses, treatments, medications, etc; (3) application submission date; (4) application review (decision) date; and (5) the outcome of the application (accepted or not accepted). This study followed the Transparent Reporting of a Multivariable Prediction Model for Individual Prognosis or Diagnosis (TRIPOD) reporting guideline. The institutional review board of Harvard University approved this study as well as the overarching UDN protocol. All UDN studies, including this one, required the patients’ or their legal guardians’ initial electronic/remote or verbal consent for participation as a research subject, which was obtained in a manner consistent with the Common Rule requirements. No one received compensation or was offered any incentive for participating in this study.

We developed classifiers using the UDN data set. The primary outcomes were whether a patient was accepted or not accepted to the UDN and a ranked list of applications based on their likelihood of admission. We partitioned the UDN data set into training (80%), validation (10%), and test (10%) data splits for retrospective evaluation (Figure 1, top box), in which the 3 splits had balanced proportions of admission labels as the UDN data set. In addition, the best classifier was prospectively tested on another 288 applications that were under review at the time of classifier development (Figure 1, middle box). The performance of the classifier was assessed using sensitivity, specificity, and area under the receiver operating characteristic curve (AUROC) metrics and by measuring the improvement in the mean processing time for accepted applications.

### Classification

We developed a logistic regression classifier with a linear kernel implemented in scikit-learn toolkit^15^ and used a grid search to optimize its hyperparameters using validation data. In addition, the classifier was trained using the following features obtained or extracted from application materials: “baseline” features obtained from patient demographic information, including normalized age at the time of application, age at disease onset, disease duration, and number of prior UDN visits. Walley et al^16^ added to the baseline features a list of objective and subjective symptoms^17^ that were manually identified in referral letters in earlier studies, where subjective symptoms were defined as patient-reported symptoms difficult to be ascertained by physical examination or medical tests (see supplementary materials in Walley et al^16^). “Referral letter” added to the features Term Frequency–Inverse Document Frequency^18^ weighted bigrams extracted from referral letters. “Semantic types” added to the features the semantic types of the Unified Medical Language System,^19,20,21^ which are a set of broad subject categories that provide a consistent categorization of medical terms; we used a select subset of semantic types (sign or symptom; laboratory, therapeutic, preventive, or diagnostic procedures; disease or syndrome; body parts or organ components; and gene or genome) as features and weighted them based on their presence (1.0) or absence (0.0) in referral letters (eTable 1 in the Supplement). “Clinical BERT” added to the features vector-based representation of referral letters obtained from the state-of-the-art language representation model called Bidirectional Encoder Representations from Transformers (BERT), which was trained on clinical text.^22^ “Online Mendelian Inheritance in Man (OMIM) similarity” added to the features cosine similarities between the clinical BERT representation of each letter and clinical BERT representations of more than 8000 phenotype entries in the OMIM database.^23^ We considered only OMIM phenotype entries, and for each entry, we used its general description and clinical features as its textual description.

### Rapid Review Process

We used the confidence score of the logistic classifier (signed distance of each data point or application to the separating hyperplane) to rank applications based on their likelihood of acceptance (Figure 1, bottom box). To obtain confidence scores for all applications, we conducted *k*-fold (*k* = 5) cross-validation experiments with the UDN data set and stored classifier confidence scores on test instances (in each fold) for final ranking purposes. Given the resulting rank list of all applications, we then measured the effect of the classifier in accelerating admission to the UDN. Specifically, we used UDN’s review session frequency and the number of applications that could be reviewed in each session to measure the mean application processing time (difference between application submission and review times) that resulted from the ranked list of applications. The process of assigning patient applications to review sessions is described in eAppendix 1 in the Supplement. We considered 4 approaches to generate ranked lists of applications: (1) UDN’s order, that is, the current processing order at the UDN; (2) first-in-first-out (FIFO) queue, meaning applications ranked based on their submission dates, with no judgment made regarding the priority of an application; (3) classifier ranking, which ranked applications based on their likelihood of acceptance generated by our best classifier; and (4) accepted-first ranking, in which applications were ranked based on their true admission labels so that the accepted and not accepted applications were separately ranked in FIFO order, and then a final ranked list of applications was generated by concatenating the 2 lists.

We note that the accepted-first ranking gives a lower bound of admission processing time for accepted applications but results in a long wait for those who are not accepted to the network. This challenge is addressed in the Discussion.

In addition, we explored how realistic resource constraints would affect the waiting times in queues for accepted and not accepted applications using the 4 application ranking heuristics. Assuming that review sessions by the UDN occur every *d* days, and that resources constrain the number of applications that can be reviewed in each session to *a* applications, we sought to determine the optimal values for *d* and *a* so that the ranking generated by our best classifier and the accept-first ranking led to minimal difference in mean processing time.

### Statistical Analysis

All tests, including *t* tests and χ^2^ tests, were 2-tailed and were conducted with SciPy 1.2.3 (a free and community-maintained toolkit for scientific computing in Python).^24^ In addition, Bonferroni adjustments were used for the χ^2^ test to measure significant difference across admission categories. Statistical significance was set at either *P* < .01 or *P* < .05.

## Results

A summary of the UDN data set is given in Table 1. The results showed that the accepted cohort was significantly younger (mean [SD] age at application, 19.7 [18.7] vs 36.5 [21.1] years) and had earlier onset of disease (mean [SD] age at symptom onset, 10.8 [16.9] vs 28.4 [21.0] year) than the not accepted cohort. The duration of disease (minimum duration, 8.89 [9.55] vs 8.12 [9.58] years) and number of prior UDN site visits (0.49 [0.56] vs 0.30 [0.68]) in both groups were comparable. However, the application processing time was significantly longer for applications that were not accepted (3.29 [3.17] vs 4.73 [4.85] months). Neurologic (575 [47.6%] and 413 [34.1%]) and musculoskeletal symptoms (151 [12.5%] and 117 [9.6%]) identified a relatively large number of applications in both groups, whereas only a few applications in each group were identified by gynecologic (2 [0.2%] and 2 [0.2%]) or toxicologic (0 [0%] and 5 [0.4%]) symptoms. The last 4 rows in Table 1 report only symptoms with a significant difference across admission categories based on Bonferroni-adjusted *P* values for the χ^2^ test.

**Table 1.** Clinical and Demographic Characteristics of Patients With Accepted vs Not Accepted Applications

Indicator	Mean (SD)		Statistics
	Accepted (n = 1209)	Not accepted (n = 1212)	
Age at application, y	19.7 (18.7)	36.5 (21.1)	*t* = −20.7^a^
Age at symptom onset, y	10.8 (16.9)	28.4 (20.1)	*t* = −22.6^a^
Minimum duration of disease, y	8.89 (9.55)	8.12 (9.58)	*t* = 2.0^b^
Prior UDN visits	0.49 (0.56)	0.30 (0.68)	*t* = 7.4^a^
Application processing time, mo	3.29 (3.17)	4.73 (4.85)	*t* = −8.6^a^
Symptoms, No. (%)			
Neurologic	575 (47.6)	413 (34.1)	χ^2^ = 144.9^a^
Musculoskeletal	151 (12.5)	117 (9.6)	
Allergic	59 (4.9)	94 (7.8)	
Gastroenterologic	47 (3.9)	92 (7.6)	
Rheumatologic	35 (2.9)	91 (7.5)	
Cardiologic	54 (4.5)	29 (2.4)	
Endocrinologic	27 (2.2)	45 (3.7)	
Pulmonologic	26 (2.2)	23 (1.9)	
Hematologic	22 (1.8)	23 (1.9)	
Infectious disease	2 (0.2)	36 (3.0)	
Dermatologic	13 (1.1)	15 (1.2)	
Nephrologic	18 (1.5)	8 (0.7)	
Ophthalmologic	14 (1.2)	8 (0.7)	
Oncologic	6 (0.5)	10 (0.8)	
Dental	8 (0.7)	6 (0.5)	
Psychiatric	5 (0.4)	5 (0.4)	
Urologic	1 (0.1)	6 (0.5)	
Gynecologic	2 (0.2)	2 (0.2)	
Toxicologic	0	5 (0.4)	
Other	115 (9.5)	130 (10.7)	
NA	29 (2.4)	54 (4.5)	
Neurologic^c^	575 (47.6)	413 (34.1)	χ^2^ = 39.51^a^
Gastroenterologic^c^	47 (3.9)	92 (7.6)	χ^2^ = 16.64^a^
Rheumatologic^c^	35 (2.9)	91 (7.5)	χ^2^ = 27.67^a^
Infectious disease^c^	2 (0.2)	36 (3.0)	χ^2^ = 30.40^a^

Abbreviation: UDN, Undiagnosed Diseases Network.

^a^ *P* < .01.

^b^ *P* < .05.

^c^ Symptoms with a significant difference across admission categories based on Bonferroni-adjusted *P* values for the χ^2^ test.

### Retrospective Analysis

The performance of different classifiers in our retrospective experiments is given in Table 2. The models that fully utilized referral letters (last 4 rows in Table 2) for classification significantly outperformed baseline and Walley et al^16^ models. In particular, the semantic types model was associated with the highest sensitivity and specificity across all models. Adding clinical BERT and OMIM similarity features was not associated with significant gain over semantic types. This may be because, in contrast to embeddings that are automatically derived from word distributions in documents, semantic types are human-expert–determined labels of related medical concepts. In this application, embeddings apparently approximated these labels with less informativeness for this classification task. Overall, the best classifier obtained sensitivity of 0.843, specificity of 0.738, and AUROC of 0.844 for predicting admission outcomes. In addition, we reported highly weighted features in eTable 2 in the Supplement and compared the precision-recall performance of classifiers in the eFigure in the Supplement.

**Table 2.** Retrospective and Prospective Classification Performances of Models Using Logistic Regression and Different Types of Features

Model	Sensitivity	Specificity	Balanced accuracy	AUROC
Retrospective				
Baseline	0.826	0.656	0.741	0.785
Walley et al^16^	0.802	0.697	0.749	0.804
Referral letter	0.860	0.713	0.786	0.831^a^^,^^b^
Semantic types	0.860	0.746	0.803	0.844^a^^,^^b^
Clinical BERT	0.843	0.721	0.782	0.844^a^^,^^b^
OMIM similarity	0.843	0.738	0.790	0.844^a^^,^^b^
Prospective				
Baseline	0.761	0.695	0.728	0.762
Walley et al,^16^	0.717	0.731	0.724	0.773
Referral letter	0.739	0.773	0.756	0.817^a^^,^^b^
Semantic types	0.739	0.790	0.765	0.829^a^^,^^b^
Clinical BERT	0.739	0.743	0.741	0.827^a^^,^^b^
OMIM similarity	0.739	0.743	0.741	0.827^a^^,^^b^

Abbreviations: AUROC, area under the receiver operating characteristic curve; BERT, Bidirectional Encoder Representations from Transformers; OMIM, Online Mendelian Inheritance in Man database.

^a^ Wilcoxon signed rank test with *P* < .05 was used for testing significance against the baseline model.

^b^ Wilcoxon signed rank test with *P* < .01 was used for testing significance against the Walley et al^16^ model.

### Prospective Analysis

The results of our prospective evaluation of 288 applications are also reported in Table 2. The performances of the different models were comparable to the results for the retrospective evaluation. The only notable difference was that baseline-only features were associated with the highest sensitivity. Adding the subjective or objective sign and symptom features proposed in Walley et al^16^ improved the overall prediction performance as shown by the AUROC but reduced the sensitivity of the classifier. As in the previous analysis, the semantic types model was associated with the highest overall performance. Further analysis of the prospective test instances showed that the best classifier failed to predict admissible applications with symptoms that had higher prevalence in the training data—for example, allergic (sensitivity = 0.4, training prevalence = 4.9%) or rheumatologic (sensitivity = 0.0, training prevalence = 2.9%) symptoms—yet perfectly predicted admitted applications for some low-prevalence symptoms—for example, hematologic (sensitivity = 1.0, training prevalence = 1.8%) or ophthalmologic (sensitivity = 1.0, training prevalence = 1.2%) symptoms (eTable 3 in the Supplement).

### Rapid Review Analysis

The mean application processing time for the 4 ranking heuristics is reported in Table 3. The results showed that our classifier could in theory reduce the mean (SD) processing time for accepted applications by approximately 68%, from 3.29 (3.17) months obtained from UDN’s current processing order to 1.05 (3.82) months by effectively prioritizing applications based on their likelihood of acceptance.

**Table 3.** Model Performance in Terms of Mean Application Processing Time

Model	Mean (SD), mo	
	Accepted	Not accepted
FIFO	3.99 (1.08)	4.10 (0.74)
UDN order	3.29 (3.17)	4.73 (4.85)
Classifier ranking	1.05 (3.82)	7.04 (10.53)
Accept-first ranking	0.28 (0.79)	7.81 (10.66)

Abbreviations: FIFO, first-in-first-out (applications ranked based on submission dates); UDN, Undiagnosed Diseases Network.

**Figure 2.**
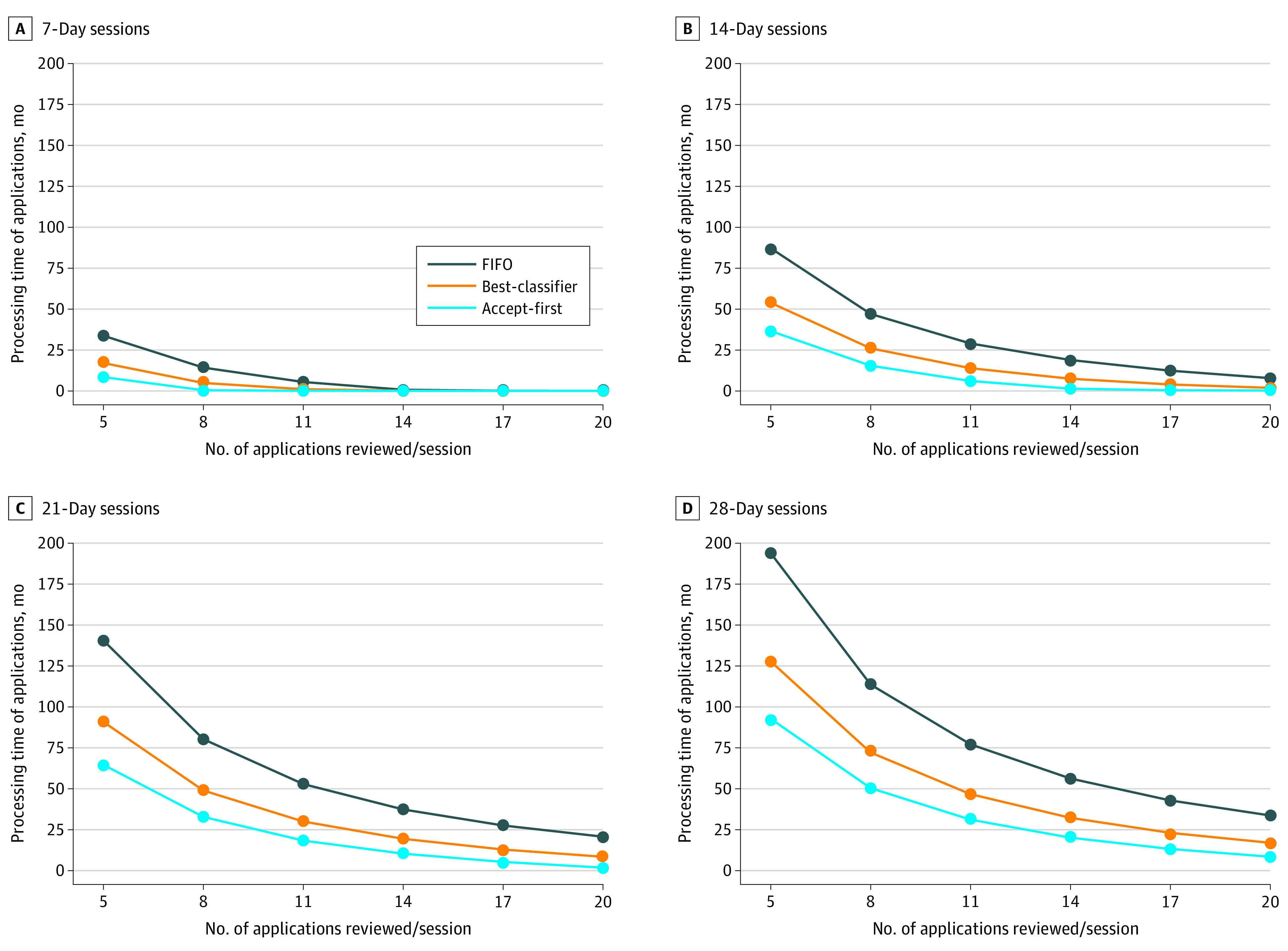
Mean Processing Times Across Different Review Periods and Number of Applications Reviewed at Each Session (Budget) FIFO represents first-in-first-out.

In addition, the results in Figure 2 showed a smaller difference in mean processing time among FIFO, our classifier, and accept-first ranking for smaller review periods (*d*) and greater resources (*a*, the number of applications reviewed per session). However, the gap increased substantially for longer review periods and smaller number of applications, a more realistic scenario. Overall, compared with the FIFO model, our classifier showed mean processing times that were closer to that of accept-first ranking across different periods and budgets. In addition, we know from empirical data that UDN review sessions were mostly organized biweekly. Our results showed that 26 applications should be reviewed at each biweekly session for our classifier (12 days) and accept-first ranking (6 days) to have comparable mean processing times (eTable 4 in the Supplement).

## Discussion

We conducted a retrospective and a prospective prognostic study to determine how well a machine learning program reproduced the performance of clinicians with expertise in genetics in determining whether to accept a patient for an extensive evaluation, including genome-scale evaluations of the patient and family members. In using the decisions of the UDN experts across 12 clinical sites in the US as the criterion standard, we found that the textual content of referral letters markedly improved the performance of machine learning programs to accurately reproduce expert decisions in both retrospective and prospective studies relative to only including the codified data (eg, demographic characteristics and manually entered Human Phenotype Ontology terms). Furthermore, including semantic types from the Unified Medical Language System further improved performance in both retrospective and prospective studies relative to purely statistical characterizations of textual content. In the prospective portion of this study, the ranking of the best machine learning model was stable, although the overall accuracy was decreased relative to the retrospective analysis.

The prospective analysis showed that although there was an enrichment for acceptance in the categories of neurology, gastroenterology, rheumatology and infectious disease, it was the rare findings that individually contributed most to the specificity of predicting an accept decision. In addition, as in any machine learning procedure, differences in the populations in the retrospective vs prospective studies (eg, due to unmeasured changes in practice of the UDN or different yields on outreach to the community by UDN staff) also contributed to a small but significant decrease in performance. Moreover, the best classifier failed to predict some accepted applications from patients with symptoms that had higher prevalence in the training data. We conjecture that more common symptom categories definitionally cover a larger variety of mechanistic etiologies and therefore give the human experts less specific evidence to believe that they can determine a novel mechanism for undiagnosed disease.

We asked how having a better sense of which patients would be admitted to the UDN might change how these patients are queued for evaluation. When we simulated knowing the experts’ decision in advance, given the limited number of application reviews that could be performed in each session, the best review frequency would be every 2 weeks. Moreover, the overall time to evaluate the accepted patients would be reduced if those that were eventually accepted (accepted-first model) were reviewed first. For example, in the FIFO model, the total time for evaluating accepted patient applications was 3.99 months vs 0.28 months in the accepted-first model with experts as the unfailing oracle. The best machine learning model to predict acceptance would result in a gain of 2.94 months in that same population (compared with the FIFO model).

### Limitations

There is unfairness in the accept-first heuristic: patients who are not going to be admitted to the UDN will have to wait the longest, only to then be told they will not be evaluated. Even if the algorithmic predictor of the experts’ eventual accept decision were perfect and the patients who were not accepted were informed immediately without additional evaluation, not accepted patients would appropriately feel unheard and uncared for. Providing a parallel process for referring these patients who received no diagnosis to other clinicians would require a thoughtful follow-up and clinical referral process depending on the reason the patient was not accepted.

In addition, there was no evaluation of the quality of the decisions made by the review committee of the UDN, such as a third-party review committee. However, we note that the UDN’s review committee consists of a group of clinicians from tertiary medical centers (having extensive experience with rare diseases and with patients who have not received a diagnosis) who critically discuss and review each application and conduct prolonged discussions for questionable cases to enable accurate admission decisions. In practice, this is at the high end, in terms of time spent and number of multidisciplinary individuals involved in clinical referral decisions. Therefore, the use of machine learning on these data represents learning from best practices and highly experienced clinical decision-makers.

Finally, the use of algorithms to decide to accept a patient to the UDN may appear to be a far-fetched and improbable scenario. Nonetheless, in the broader practice of medicine, the decision to make or allow referrals to a different clinician, health care system, or specific high-cost evaluation is now transitioning from human review (itself the cause of some dissatisfaction and concern^25^) to an automated process driven by algorithms.^26^ This process happens largely within the commercial sphere and without scientific peer review. The evaluation process that we describe here would at a minimum give clinicians and patients better insight into this gatekeeping process.

## Conclusions

We described a machine learning approach to predicting expert decisions on accepting patients to the UDN based on the materials provided by patients and their clinicians. The best machine learning models we developed predicted admission outcomes with an AUROC of 84.4% and in theory accelerated the admission process for accepted applications by 68%. Such a shorter turnaround time could potentially improve diagnosis journeys for most patients with undiagnosed diseases. It could also reduce the overall cost of diagnosis because the longer it takes to accept patients, the more (costly) diagnostic routes are likely to be sought by them.^6^ Incorporating such an automated approach requires rethinking the workflow of evaluating patients and particularly considering safely and efficiently managing cases not accepted into the network. In considering these possibilities, we hope to stimulate discussion of current practices of automating referral decisions in the broader context of health care.

## Notes

Supplement. **eTable 1.** Normalized Term Frequency of Several Semantic Types in the Referral Letters of Accepted and Not-Accepted Applications **eTable 2.** Top Features and Their Corresponding Weights for Accepted and Not-Accepted Application Classes **eFigure.** Comparison of Different Models in Terms of Their Ranking Performance Illustrated by Precision-Recall Curve **eTable 3.** Symptom-Level Performance on Prospective Test Instances **eAppendix 1.** Process for Assigning Patient Applications to Review Sessions **eTable 4.** Average Processing Time Across Different Review “Periods” and Number of Applications Reviewed in Each Review Session (“Budgets”) **eAppendix 2.** Data Set
