# Supplementary material for: Machine Learning of Patient Characteristics to Predict Admission Outcomes in the Undiagnosed Diseases Network
Source: JAMA Netw Open. 2021 Feb 25;4(2):e2036220. doi: 10.1001/jamanetworkopen.2020.36220 (PMC7907957; doi:10.1001/jamanetworkopen.2020.36220)
Supplement: Supplement. — eTable 1. Normalized Term Frequency of Several Semantic Types in the Referral Letters of Accepted and Not-Accepted Applications eTable 2. Top Features and Their Corresponding Weights for Accepted and Not-Accepted Application Classes eFigure. Comparison of Different Models in Terms of Their Ranking Performance Illustrated by Precision-Recall Curve eTable 3. Symptom-Level Performance on Prospective Test Instances eAppendix 1. Process for Assigning Patient Applications to Review Sessions eTable 4. Average Processing Time Across Different Review “Periods” and Number of Applications Reviewed in Each Review Session (“Budgets”) eAppendix 2. Data Set [file jamanetwopen-e2036220-s001.pdf]

Supplementary Online Content

Amiri H, Kohane IS; Undiagnosed Diseases Network. Machine learning of patient characteristics to predict admission outcomes in the Undiagnosed Diseases Network. *JAMA Netw Open*. 2021;4(2):e2036220. doi:10.1001/jamanetworkopen.2020.36220

eTable 1. Normalized Term Frequency of Several Semantic Types in the Referral Letters of Accepted and Not-Accepted Applications
eTable 2. Top Features and Their Corresponding Weights for Accepted and Not-Accepted Application Classes
eFigure. Comparison of Different Models in Terms of Their Ranking Performance Illustrated by Precision-Recall Curve
eTable 3. Symptom-Level Performance on Prospective Test Instances
eAppendix 1. Process for Assigning Patient Applications to Review Sessions
eTable 4. Average Processing Time Across Different Review "Periods" and Number of Applications Reviewed in Each Review Session ("Budgets")
eAppendix 2. Data Set

This supplementary material has been provided by the authors to give readers additional information about their work.

© 2021 Amiri H, et al. *JAMA Network Open*.

eTable 1. Normalized Term Frequency of Several Semantic Types in the Referral Letters of Accepted and Not-Accepted Applications

UMLS contains a set of broad subject categories, or semantic types, that provide a consistent categorization of all medical terms/concepts represented in the UMLS Metathesaurus. We counted the frequency of a select subset of these semantic types in the referral letters of Accepted and Not-accepted applications using our "training" data. The results are reported in **eTable 1**.

Results are obtained from training data. SD indicates Standard Deviation and t indicates t-statistic of two-tailed t-test with *p<0.05 and ***p<0.001.

**Semantic Type**	**Accepted (mean±SD)**	**Not-accepted (mean±SD)**	**Statistics**
**Sign or symptom**	**19.41±0.061**	**33.62±0.14**	**t = -1.98***
**Clinical drug**	**0.36±0.00**	**0.84±0.00**	**t = -6.46*****
**Laboratory procedure**	56.49±0.23	51.78±0.21	t = 0.29
**Disease/syndrome**	109.07±0.26	105.03±0.22	t = 0.12
**Therapeutic procedure**	44.60±0.28	44.35±0.28	t = 0.01
**Body part organ**	120.10±0.46	115.97±0.43	t = 0.11
**Gene or genome**	198.22±0.63	186.45±0.59	t = 0.18
**Diagnostic procedure**	23.34±0.10	22.53±0.10	t = 0.14

The results show that laboratory procedure, disease or syndrome, therapeutic and preventive procedure, body part organ, gene or genome, and diagnostic procedures more frequently occur in Accepted applications. This is while signs and symptoms and clinical drugs more frequently occur in Not-accepted applications.

© 2021 Amiri H, et al. *JAMA Network Open*.

eTable 2. Top Features and Their Corresponding Weights for Accepted and Not-Accepted Application Classes

Classification algorithms learn coefficients (weights) for features in order to make predictions. These coefficients can be used directly as a type of feature importance score. We reported highly weighted features for both Accepted and Not-accepted applications in **eTable 2**

Accepted Application Class		Not-accept Application Class	
**Feature**	**weight**	**Feature**	**weight**
sensorineural hearing loss of both ears	1.23	p30	-2.25
generalized abdominal pain	1.04	sweat gland	-1.47
excessive sleeping	0.97	fancm	-1.26
bile acid	0.95	alp	-0.79
dissection	0.8	lewy body disease	-0.78
gavage	0.77	transitional epithelial cells	-0.68
amp	0.63	hdac8	-0.64
proband	0.6	blood sugar monitoring	-0.63
xomedx	0.6	tenderness eye	-0.61
lipase deficiency	0.59	nasal cpap	-0.55
myl	0.59	dissection neck	-0.54
aspm	0.51	chemotherapy	-0.47
strc gene	0.51	rasmussen encephalitis	-0.47
external auditory canals	0.47	polymorphism	-0.46
multiple chemical sensitivity	0.47	demanding	-0.45
multiple congenital	0.45	abcb11	-0.44
ofd1 gene	0.45	brucella antibody	-0.43
behavior	0.43	mbp	-0.42
gu	0.43	transport media	-0.41
generalized pain	0.42	gallium scan	-0.4
spasm muscle	0.42	spherocytes	-0.4
articulation	0.41	alkalosis	-0.39
causative	0.41	brain ct	-0.38
spine normal	0.41	discuss	-0.38

© 2021 Amiri H, et al. *JAMA Network Open*.

il6	0.4	microarray analysis	-0.38
central tegmental tracts	0.39	diabetes mellitus without complication	-0.37
pain tenderness	0.38	flexor	-0.37
secondary hyperparathyroidism	0.38	ammonia blood	-0.35
seen rheumatology	0.37	fibrin split products	-0.35
sgpt	0.37	hair loss	-0.35
transthoracic echocardiography	0.36	methadone maintenance	-0.35
generalized erythroderma	0.35	tgm6	-0.35
crowning	0.32	hco3	-0.34
cp24	0.31	indirect ophthalmoscopy	-0.34
radiographic imaging	0.31	pylorus	-0.34
dependent rubor	0.3	vitb12	-0.34
cachexia	0.29	zyrtec	-0.34
endocrine nutritional metabolic disorder	0.28	congenital rubella	-0.33
fibroadenosis of breast	0.28	fevers recurrent	-0.33
disorder genetic	0.27	tetrahydrobiopterin deficiency	-0.33

© 2021 Amiri H, et al. *JAMA Network Open*.

eFigure. Comparison of Different Models in Terms of Their Ranking Performance Illustrated by Precision-Recall Curve

We compared classification models in terms of their ability to rank Accepted applications above Not-accepted ones. The **eFigure** shows the precision-recall curve for Walley et al. (2018)[16] and our two BERT-based models (for clear illustration, we only showed the results of these three models). The no-skill line was a straight line at precision = 0.5. The results showed BERT-based models were considerably more precise (able to correctly rank Accepted applications) at lower recall values.

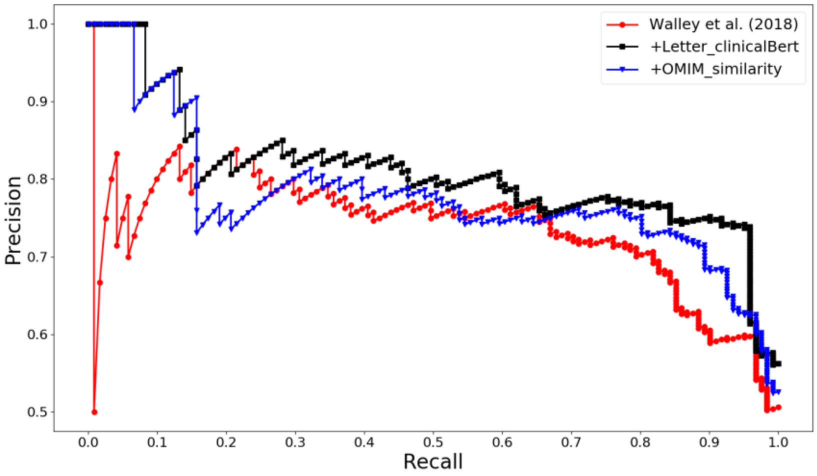

© 2021 Amiri H, et al. *JAMA Network Open*.

eTable 3. Symptom-Level Performance on Prospective Test Instances

eTable 3 illustrates symptoms for which our best model succeeded (green) or failed (yellow) in predicting admission outcomes on the prospective test instances. %preval indicates prevalence in training of a symptom in training/test data.

	Training	Test					
	%preval	%preval	%accepted	%not_accepted	Accuracy	sensitivity	specificity
**Neurology**	42.3	40.8	45.2	54.8	0.7500	0.8298	0.6842
**Musculoskeletal**	11.4	11.8	53.3	46.7	0.7000	0.7500	0.6429
**Other**	10.2	12.2	16.1	83.9	0.8710	0.6000	0.9231
**Allergies**	6.6	7.5	26.3	73.7	0.7368	0.4000	0.8571
**Gastroenterology**	6.3	7.1	27.8	72.2	0.8889	0.6000	1.0000
**Rheumatology**	5.4	5.5	21.4	78.6	0.7857	0.0000	1.0000
**Cardiology**	3.7	4.3	27.3	72.7	0.5455	0.6667	0.5000
**Endocrinology**	3.0	3.1	25.0	75.0	0.7500	0.5000	0.8333
**Pulmonology**	2.0	0.8	0.0	100.0	0.0000	-	0.0000
**Hematology**	1.9	2.4	16.7	83.3	0.8333	1.0000	0.8000
**Infectious**	1.6	0.8	0.0	100.0	1.0000	-	1.0000
**Dermatology**	1.2	0.0	-	-	-	-	-
**Nephrology**	1.1	0.4	100.0	0.0	1.0000	1.0000	-
**Ophthalmology**	1.0	1.2	66.7	33.3	1.0000	1.0000	1.0000
**Oncology**	0.7	0.8	0.0	100.0	1.0000	-	1.0000
**Dentistry**	0.6	1.2	33.3	66.7	1.0000	1.0000	1.0000
**Psychiatry**	0.4	0.4	100.0	0.0	0.0000	0.0000	-
**Urology**	0.3	0.0	-	-	-	-	-

© 2021 Amiri H, et al. *JAMA Network Open*.

**Toxicology**	0.2	0.0	-	-	-	-	-
**Gynecology**	0.2	0.0	-	-	-	-	-

© 2021 Amiri H, et al. *JAMA Network Open*.

eAppendix 1. Process for Assigning Patient Applications to Review Sessions

Given a ranking heuristic, we follow the following process to assign applications to review sessions: each review session i in the UDN dataset had a meeting date t_i and a budget b_i which indicates the number of applications that could be reviewed at that review session. Given the review sessions in ascending order of their meeting dates $t_i \ \forall i=1,2,..,$ and a ranked list of applications in descending order of their admission likelihood, we assigned the top b_i applications that were submitted before date t_i to the ith review session (and set their review/decision dates to t_i). These top b_i applications were then removed from the ranked list of applications and this process was repeated for subsequent review sessions until all applications were assigned to review sessions. This process ensured that applications were assigned to their closest review sessions while respecting the budget/time constraint for each session and the order of applications in the given ranked list of applications. Once all applications were assigned review dates, we computed the average processing time by computing the time difference between application submission and review dates as follows: $\sum_{j=1}^{n} (r_j - s_j)$where s_j and r_j were the submission and assigned review dates of the jth application. Note that information about meeting dates and budget for each session was provided by the UDN.

© 2021 Amiri H, et al. *JAMA Network Open*.

eTable 4. Average Processing Time Across Different Review "Periods" and Number of Applications Reviewed in Each Review Session ("Budgets")

Let *d* be the review frequency (i.e. review sessions occur every *d*-day period) and *a* be the number of applications reviewed in each session (budget for each session). The optimal values for *d* and *a* so that the ranking generated by our best classifier and the Accept-First ranking model lead to the same/comparable (i.e. a difference of less than a week) average processing time are highlighted in **eTable 4**. For example, for biweekly review sessions (d = 14), at least a = 26 applications should be reviewed at each session so that our best classifier leads to a comparable processing time to the Accept-First model.

We look for combinations in which the ranking generated by the classifier and the perfect ranking lead to the same/comparable average processing time. The highlighted rows show periods and budgets for which our classifier and perfect model have a maximum difference of one week.

Period	Budget	FIFO (Months)	Classifier (Months)	Perfect (Months)
7	2	114	72.57	50.8
7	5	33.73	17.18	8.45
7	8	14.26	4.98	0.43
7	11	5.47	1.13	0.1
7	14	0.74	0.17	0.09
7	17	0.13	0.09	0.09
7	20	0.09	0.09	0.09
7	23	0.09	0.09	0.09
7	26	0.09	0.09	0.09
7	29	0.09	0.09	0.09
7	32	0.09	0.09	0.09
7	35	0.09	0.09	0.09
7	38	0.09	0.09	0.09
7	41	0.09	0.09	0.09
7	44	0.09	0.09	0.09
7	47	0.09	0.09	0.09
7	50	0.09	0.09	0.09
7	53	0.09	0.09	0.09
7	56	0.09	0.09	0.09
7	59	0.09	0.09	0.09
14	2	247.67	165.07	121.26
14	5	87.12	53.92	36.56
14	8	46.99	26.21	15.39
14	11	28.95	13.92	6.05
14	14	18.88	7.58	1.34
14	17	12.36	4.08	0.38
14	20	7.8	2.04	0.23
14	23	4.46	0.98	0.2
14	26	1.91	0.4	0.2
14	29	0.61	0.25	0.2
14	32	0.31	0.21	0.2
14	35	0.23	0.21	0.2
14	38	0.21	0.2	0.2
14	41	0.2	0.2	0.2
14	44	0.2	0.2	0.2

© 2021 Amiri H, et al. *JAMA Network Open*.

14	47	0.2	0.2	0.2
14	50	0.2	0.2	0.2
14	53	0.2	0.2	0.2
14	56	0.2	0.2	0.2
14	59	0.2	0.2	0.2
21	2	381.33	257.52	191.73
21	5	140.52	90.85	64.68
21	8	80.32	49.17	32.92
21	11	52.96	30.29	18.48
21	14	37.47	19.64	10.47
21	17	27.69	13.08	5.46
21	20	20.84	8.91	1.95
21	23	15.77	5.88	0.87
21	26	11.88	3.89	0.45
21	29	8.8	2.45	0.35
21	32	6.29	1.48	0.32
21	35	4.21	0.98	0.32
21	38	2.47	0.59	0.32
21	41	1.1	0.41	0.32
21	44	0.65	0.35	0.32
21	47	0.44	0.33	0.32
21	50	0.36	0.32	0.32
21	53	0.34	0.32	0.32
21	56	0.33	0.32	0.32
21	59	0.32	0.32	0.32
28	2	515	349.94	262.2
28	5	193.92	127.77	92.8
28	8	113.65	72.2	50.45
28	11	77.16	46.95	31.2
28	14	56.31	32.6	20.2
28	17	42.92	23.38	13.3
28	20	33.75	17.2	8.56
28	23	26.96	12.55	5.06
28	26	21.75	9.46	2.4
28	29	17.63	6.95	1.26
28	32	14.35	5.17	0.75
28	35	11.62	3.8	0.57
28	38	9.32	2.76	0.49
28	41	7.37	2.03	0.45
28	44	5.69	1.42	0.44
28	47	4.21	1.07	0.44
28	50	2.92	0.79	0.44
28	53	1.77	0.58	0.44
28	56	1.07	0.52	0.44
28	59	0.75	0.47	0.44

© 2021 Amiri H, et al. *JAMA Network Open*.

eAppendix 2. Data Set

The original size of the dataset was 2,518 applications. We discarded applications for which we couldn't extract the textual content of the referral letters; these are mainly applications that submitted images (or in few cases video clips) of referral letters that led to poor Optical Character Recognition (OCR) quality. We note that to improve patient and referring clinician convenience, UDN accepts all formats for referral letters. We manually checked the OCR output for all referral letters to identify poor OCR outputs and discarded corresponding applications.
